# Advanced Silica Gel/Sulfonated Polymer Composites for Electric Vehicle Thermal Management by Sorption Technology

**DOI:** 10.3390/ma19030625

**Published:** 2026-02-06

**Authors:** Davide Palamara, Mengistu Gelaw, Emanuela Mastronardo, Andrea Frazzica, Candida Milone, Luigi Calabrese

**Affiliations:** 1 Department of Engineering, University of Messina, Contrada di Dio Sant’Agata, 98166 Messina, Italy; mengistugelaw.woyesa@unime.it (M.G.); emanuela.mastronardo@unime.it (E.M.); candida.milone@unime.it (C.M.); luigi.calabrese@unime.it (L.C.); 2 Department of Mechanical Engineering, School of Mechanical, Chemical and Materials Engineering, Adama Science and Technology University, Adama 1888, Ethiopia; 3 CNR ITAE “Nicola Giordano”—Institute of Advanced Technologies for Energy, Via Salita S. Lucia Sopra Contesse 5, 98126 Messina, Italy; andrea.frazzica@cnr.it

**Keywords:** sulfonated polymer, silica gel, coating, adsorption, adhesion, water vapor, heat management

## Abstract

This study explores novel silica gel/sulfonated polymer composite coatings for enhanced thermal management in electric vehicles via sorption technology. Leveraging the cost-effectiveness of silica gel as a filler and a readily available, water vapor-permeable sulfonated polymer as the matrix, we developed and characterized these materials. Mechanical assessments revealed varied performance: coatings with lower silica gel content (80 and 85 wt%) demonstrated suitable scratch resistance (damage width ~1100 µm at 1300 g load) and superior impact resistance (damage diameter ~2.4 mm). Pull-off adhesion strengths for these batches were 1.26 MPa and 1.36 MPa, respectively, though higher filler loading (90 and 95 wt%) led to a ~30% reduction and a shift to cohesive failure for high-filler-content batches. Thermogravimetric analysis confirmed thermal stability up to 280 °C. Adsorption studies revealed that the composite coating with 95 wt% of silica gel achieved the highest water uptake (just under 30 wt%), with all batches exhibiting capacities comparable to commercial adsorbents. This comprehensive characterization confirms that these composites offer a compelling balance of mechanical robustness, reliable adhesion, and high adsorption efficiency, positioning them as promising, cost-effective solutions for EV thermal management.

## 1. Introduction

The rapid global transition towards electric vehicles (EVs) as a cornerstone of sustainable transportation necessitates significant advancements across various technological domains, with thermal management emerging as a critical frontier [1]. Unlike conventional internal combustion engine vehicles, EVs demand sophisticated energy management to optimize performance, enhance durability, and improve the overall affordability of the vehicles [2,3]. Maintaining the battery within a narrow optimal temperature window (typically 20–35 °C) is paramount to preventing degradation, ensuring efficient charging and discharging, and mitigating the risk of thermal runaway [4,5]. Beyond powertrain components, ensuring passenger comfort through effective cabin heating and cooling also presents a substantial challenge, often leading to a reduction in crucial driving range due to the energy demands of conventional vapor-compression HVAC systems [6]. These challenges underscore the urgent need for innovative, energy-efficient, and environmentally benign thermal management solutions for the next generation of electric vehicles [7,8].

Sorption technology offers a sustainable, energy-efficient thermal management alternative, crucial for electric vehicles [9]. It uses low-grade waste heat to drive the reversible adsorption of refrigerants, typically water, onto solid materials [10,11]. The inherent advantages of sorption systems, including their environmental compatibility, low energy consumption, and high safety profile, position them as highly promising candidates for integrated thermal management in electric vehicles, encompassing both battery thermal regulation and cabin climate control.

Traditional solid adsorbents often exhibit limitations in terms of adsorption capacity, sorption kinetics, and mechanical stability [12,13]. To overcome these challenges, the development of composite adsorbent coatings has attracted considerable attention. These composites typically consist of an adsorbent material (filler) dispersed within a polymer matrix (binder), which enhances mechanical integrity, improves heat and mass transfer [14,15,16], and facilitates direct application onto heat exchanger surfaces [12,17]. Consequently, the choice of both the adsorbent filler and the polymer matrix is pivotal to achieving the desired performance and cost-effectiveness.

In this regard, a new energy-efficient automotive AC system, the desiccant-coated heat exchanger (DCHE) is designed to reduce humidity by using a low-grade heat source. Through this approach, a heat exchanger coated with desiccant material absorbs or releases moisture as fluid passes through [18]. Zhang et al. [19] reported a 42% reduction in cabin heat load and 38% decrease in compressor electric power in an EV AC system using a DCHE. Analogous considerations were argued by Zadeh et al. [20], who proposed a new integrated desiccant-coated heat pump (DCHP) that improves efficiency by eliminating the need for an additional heat exchanger, thereby enhancing EV thermal management significantly. Na et al. [21] further demonstrated a DCHE’s ability to create more efficient AC with lower humidity and optimal temperatures. However, a key challenge is continuous operation without adding weight, which could reduce EV cruising range.

While various adsorbents, such as zeolites (e.g., 13X and SAPO-34) [19,22,23], salt-hydrate [24], or metal–organic frameworks (MOFs) [25,26], have been explored for adsorption technologies, silica gel stands out as an economically attractive option [20]. Its lower cost compared to most zeolites and MOFs makes it particularly appealing for large-scale applications, despite other materials potentially offering higher theoretical adsorption capacities [27]. To ensure long-term durability in transportation, where heightened vibrations are a constant, sorbent coatings must be mechanically stable; thus, integrating cost-effective adsorbents into a robust and functional coating necessitates a suitable polymer matrix.

In this context, Calabrese et al. [28] investigated zeolite–polymer composite coatings, determining that an 80 wt% filler content offered the best balance of mechanical stability and adsorption capacity. This research activity also underscored the potential of silane SAPO-34 composites for enhancing power density in adsorption systems.

Therefore, the dual functionality of a matrix, exhibiting both structural resilience under thermal stress and superior moisture transport properties, is a critical advancement that addresses the typical trade-offs in adsorbent coating design [29]. Such a matrix not only needs to ensure the structural integrity of the coating during repeated thermal adsorption–desorption cycles but will also be able to facilitate rapid mass transfer to the active filler, making it a highly promising candidate for high-efficiency sorbent applications.

In this regard, sulfonated polymers emerge as a promising class of binders due to their inherently open structure and hydrophilic nature, which actively facilitate the efficient transport of water vapor throughout the composite. Sulfonated polyether-ether ketone (S-PEEK) matrix, owing to its high water vapor permeability and robust mechanical properties, has been demonstrated as a suitable candidate for adsorbent composite coatings, providing promising adsorption performance even at high filler contents [30].

Furthermore, investigations into sulfonated recycled-PEEK revealed stable sulfonic groups across various temperatures, exhibiting a satisfactory balance between mechanical properties and ad/desorption performance [31].

Despite significant progress, further research is essential for comprehensively optimizing composite coating formulations for large-scale applications, particularly regarding their long-term cyclic stability under varying environmental conditions [32]. Industrial scalability, manufacturing processes, and cost-effectiveness also require additional assessment.

Recent research emphasizes the remarkable water management properties of Nexar™, a sulfonated pentablock terpolymer, a readily available commercial product [33]. Its unique polymeric architecture efficiently facilitates water vapor diffusion, making it highly suitable for applications where water uptake capacity is required [34,35], making Nexar a promising polymer matrix for advanced adsorbent composite coatings.

Building upon these premises, the aim of this work is to evaluate the synergistic potential of a novel, high-performance composite coating engineered using readily available silica gel and sulfonated pentablock terpolymers as the binders. Unlike traditional binders, this material selection represents a strategic shift toward industrial scalability; it leverages the unique sulfonated architecture of the terpolymer to overcome the classic trade-off between mechanical robustness and mass transfer resistance. Their inherent hydrophilic nature also actively facilitates crucial water vapor transport for efficient adsorption/desorption cycles. The novelty of this approach lies in the development of a cost-effective, high-filler-loading composite that remains mechanically stable under mechanical stresses typical of demanding automotive environments. By bridging the gap between advanced polymer science and practical thermal management, this work provides a reliable solution designed to offer enhanced adsorption, superior mechanical stability, and robust substrate adhesion, all critical for ensuring electric vehicle (EV) reliability while simultaneously improving both EV performance and passenger comfort.

In this regard, the present study focuses on the initial performance and the synergistic properties of this novel coating family, establishing a fundamental baseline based on their mechanical and adsorption behaviors. Specifically, in this study, the influence of filler content on the morphological, thermal, mechanical, and functional properties of the Nexar/silica gel composite coating is systematically investigated. By balancing the high adsorption capacity of the filler with the structural integrity and high water vapor permeability provided by the sulfonated matrix, this work identifies the optimal formulation for efficient and durable sorption-based coatings.

## 2. Materials and Methods

### 2.1. Materials

The matrix for the composite coating was Nexar™, a sulfonated pentablock terpolymer from Kraton Corporation Inc. (Houston, TX, USA). Coating deposition was carried out using N,N-Dimethylformamide (N,N-DMF) (≥99.8% pure) as the solvent, sourced from Sigma Aldrich (St. Louis, MO, USA). The silica gel powder (average particle size: <25 µm) used as functional filler in the composite coating was obtained from Sorption Technologies GmbH (Freiburg, Germany).

### 2.2. Coating Preparation

**Figure 1:**
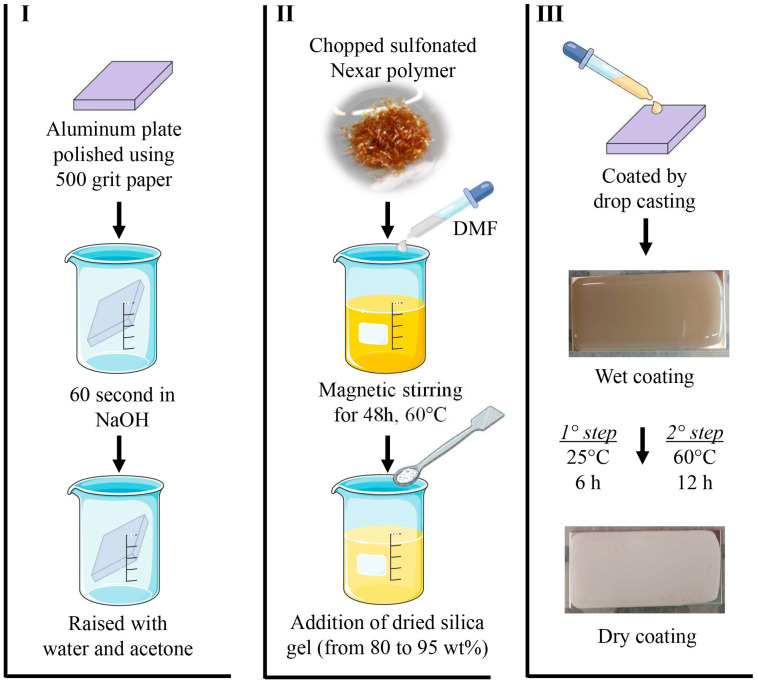
Preparation procedures of composite coating: (I) Substrate pre-treatment; (II) Composite slurry preparation; (III) Deposition and curing.

The composite coating preparation can be summarized as follows (Figure 1) in three main steps:

- An aluminum substrate measuring 40 × 20 × 2 mm was used for preparing the coating samples. The substrates were first polished using 500-grit emery paper, then subjected to a 60 s cleaning process in a 0.1 NaOH alkaline solution. Following this, they were rinsed with deionized water and acetone to remove any residual contaminants. Finally, the mass of each aluminum substrate was measured.
- Nexar polymer (SPT) was dissolved in N,N-Dimethylformamide (DMF) using a magnetic stirrer at 250 rpm for 48 h at 60 °C. The beaker was fully covered with parafilm throughout this process to prevent DMF evaporation and preserve solution viscosity. Afterward, the silica gel filler, pre-dried overnight at 120 °C, was added to the polymer solution and mixed via magnetic stirring at 60 °C and 250 rpm for one hour to achieve a homogeneous composite slurry.
- Finally, the composite slurry was applied onto the aluminum substrate using a drop-casting technique. The coated samples were allowed to cure at room temperature for three hours, followed by overnight drying at 60 °C. Subsequently, the thickness of the fully dried composite coatings was measured by means of a digital thickness gauge, with a resolution of 1 µm. For each formulation, the thickness was evaluated at six different locations per sample across three different specimens.

The composite coatings were fabricated in accordance with the methodologies previously described and validated in [36], with specific adjustments made for the current filler–matrix ratios.

With this procedure, four different batches were realized, with silica gel content ranging from 80 to 95 wt.%. All samples were designated by a prefix “NSG”, where “N” stands for Nexar (sulfonated pentablock terpolymers), and “SG” represents silica gel, followed by a number indicating the silica gel content by weight percentage. For instance, NSG80 refers to a composite containing 80 wt% silica gel and 20 wt% Nexar. As a reference, the unfilled polymer coating was also used (coded by N).

All coating deposition and subsequent room temperature drying processes were carried out under controlled laboratory conditions, maintaining an ambient temperature of 25 ± 2 °C and a relative humidity of 50 ± 5%, to ensure consistency across batches.

All manufactured samples appear macroscopically homogeneous, showing no visual evidence of phase separation, filler agglomeration, or superficial cracks. This structural integrity is important in order to ensure its suitability and reproducibility for the subsequent coating performance evaluation.

The average thickness of the composite coatings ranges from 0.76 to 0.92 mm. While fluctuations in thickness were observed, stemming primarily from the manual drop-casting process and filler-dependent slurry viscosity, the statistical overlap between batches (e.g., NSG80 and NSG85, representing the minimum and maximum average values, respectively) ensures a comparable baseline for subsequent mechanical evaluation.

The discrepancy in thickness between the composite coatings and the pure Nexar (polymer) reference is a result of their different material compositions. In the composite samples, the high filler loading (from 80 to 95 wt%) provides a structural support that influences the final thickness. Conversely, the pure polymer lacks this filler-based volume and undergoes significant volumetric shrinkage during solvent evaporation and curing, thus leading to a lower coating thickness.

Table 1 reports the sample nomenclature, the wet formulation ratios of the composite mixtures, and the final composition of the cured coatings in terms of silica gel and polymer matrix content, along with the respective coating thicknesses (in terms of average and standard deviation across all samples).

### 2.3. Composite Coating Characterization

#### 2.3.1. Mechanical Characterization

A thorough understanding of the mechanical properties and predominant failure mechanisms of the composite-coated surface is essential before its application to the HVAC systems. Due to the distinct physico-chemical and mechanical characteristics of the coating materials and their substrates, unique failure patterns often emerge. This mismatch can induce residual stresses and strains within the composite layer or at the coating–substrate interface, potentially leading to premature cracking and its propagation. To ensure long-term reliability and service life, it is crucial to evaluate the mechanical integrity of the composite, particularly under dynamic, static, or hydrothermal loading conditions associated with the adsorption–desorption cycles [25,31]. Accordingly, the procedures outlined in [29] were followed to investigate scratch resistance, pull-off adhesion strength, and impact performance as part of the mechanical characterization of the composite coating.

Scratch Test: To evaluate the adhesion characteristics of the composite coating, a scratch test was performed in accordance with the ISO 1518-1 standard [37]. The test employed an AISI 304 stainless-steel ball, shaped into a truncated cone (45° angle and 500 µm height) with a maximum radius of 450 µm mounted on a matching conical support. Incremental loads ranging from 100 to 1300 g were applied to the indenter arm, with scratch lines spaced 5 to 10 mm apart. The resulting groove widths corresponding to each applied load were then analyzed using a HK-8700 3D optical digital microscope (Hirox, Tokyo, Japan). In order to assess the result’s reproducibility, three replicates were performed for each batch.

Impact test: The primary objective of this test is to assess the composite coating’s behavior under sudden impacts from varying heights. The procedure outlined in reference [17] was followed to carry out the impact assessment. The coated specimen was positioned on a 45° inclined support to prevent rebound of the 32.7 g, 24 mm diameter metallic sphere. The sphere was initially held in place using an electromagnet and then released to free fall from different heights ranging from 10 to 50 cm. After impact, the diameters of the resulting indentations were measured using the same optical microscopy reported above, and the absorbed impact energy was calculated using the formula E = *mgh*, where *m* represents the ball’s mass, *g* is the gravitational acceleration, and *h* is the drop height. In order to assess the result’s reproducibility, three replicates were performed for each batch.

Pull-off test: A pull-off test (ASTM D 4541) [38], as described in ref. [30], was used to evaluate the composite coating’s adherence to the metal substrate. A stainless-steel dolly with a 10 mm diameter was used with a DeFelsko PosiTest AT-M tester (DeFelsko, Ogdensurg, NY, USA). Before performing the pull-off test, cyanoacrylate adhesive (shear and tensile strength ranging between 6 and 22 MPa) was used to attach the dolly to the coated surface, and it was left to cure for 12 h at room temperature in the air.

To accurately evaluate adhesion strength, it is essential to distinguish between the different fracture modes: adhesive failure, occurring at the interface between the coating and the aluminum substrate, and cohesive failure, characterized by a fracture within the bulk of the composite itself. This distinction indicates whether the structural limit lies at the coating/substrate interface or within the composite matrix, providing critical insight into the influence of filler content on coating integrity. In order to assess the result’s reproducibility, three replicates were performed for each batch.

#### 2.3.2. Thermo-Physical Characterization

Thermo-physical characterization is essential for understanding the performance and stability of silica gel/Nexar composite coatings designed for sorption-based systems. This analysis evaluates key parameters such as thermal stability, chemical interactions between silica gel particles and the Nexar polymer matrix, and water vapor sorption–desorption behavior. Collectively, these assessments reveal the relationship between material composition, thermal resilience, and sorption efficiency, supporting the optimization of composite coatings for durable and efficient applicability.

Thermal analysis: Thermal stability of the composite coatings was evaluated using a TA instruments Q600 (TA Instruments, Newcastle, DE, USA). A 5–10 mg sample was placed in an alumina crucible and heated to 120 °C at a rate of 10 °C/min, then held at that temperature for 60 min to remove moisture. Subsequently, the sample was further heated to 620 °C at the same rate under an airflow of 100 mL/min. The TGA thermal scans were focused on the range up to 620 °C to fully characterize the decomposition profile of the sulfonated polymer binder. This temperature range is representative of the total degradation of the organic fraction and far exceeds the maximum operating temperatures required for automotive thermal management applications, where the coating must remain structurally intact.

Fourier-transform infrared spectroscopy analysis: The functional groups in the prepared composite coatings were analyzed using Fourier-transform infrared spectroscopy (FTIR) with an Agilent Cary 670 spectrometer (Agilent, Santa Clara, CA, USA). Spectra were recorded at a resolution of 1 cm^−1^ over the range of 400–4000 cm^−1^. Raw Nexar, all composite coating samples, and silica gel powder were examined and compared.

Adsorption/desorption analysis: Equilibrium water vapor adsorption/desorption measurements were performed under controlled temperature and partial pressure environments by using a thermogravimetric dynamic vapor sorption system (DVS Vacuum, Surface Measurement Systems, Alperton, London, UK). This set up features a high-precision microbalance (±0.1 µg accuracy) and a vapor pressure regulation unit integrated within the measurement chamber. The hydration/dehydration cycles were performed in isothermal mode at a temperature of 30 °C with a partial pressure ranging from 0 to 90%.

Morphological analysis: To assess homogeneity and to relate the coating performance with its microstructure, a morphological analysis of the composite coating was conducted utilizing an SEM-FEI Quanta FEG (Quanta, Hillsboro, OR, USA) scanning electron microscope operated under a nitrogen environment in a high vacuum condition. For this SEM analysis, samples were fixed to aluminum stubs via a conductive adhesive film.

## 3. Results

### 3.1. Mechanical Results

The main goal of this characterization is to evaluate the composite coatings’ adhesion, as well as their impact and scratch resistance when applied to a metallic substrate. The adhesive and cohesive forces are related to the interaction between the Nexar polymer, the filler silica gel particles, and the aluminum substrate.

**Figure 2:**
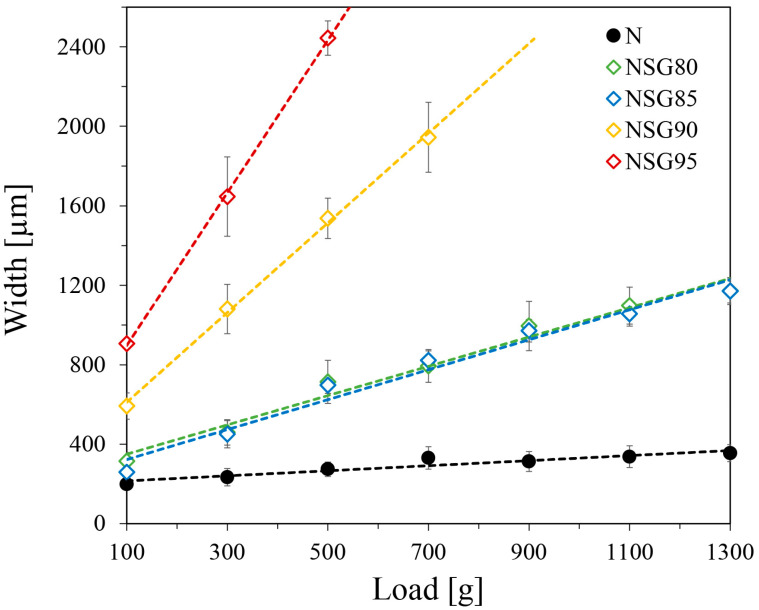
Scratch width evolution at increasing load for the unfilled and filled coatings during scratch test. The experimental data were fitted using linear curves (dashed lines) yielding R^2^ ≥ 0.97 for all tested samples. Error bars represent the standard deviation of three independent measurements.

First, concerning the scratch test, Figure 2 compares the trends in groove width as a function of applied load for various silica gel weight percentages. The unfilled polymer (N) was also added as a reference. Primarily, with the raw polymer (N), the groove width remains very low regardless of the applied load. This consistent width, evidenced by the curve’s minimal slope, implies a strong adhesive bond between the binder and the substrate, making the polymer coating highly resistant to load variations. A different trend can be observed when evaluating the composite coatings; the profile trends show a linear increase in groove width as the applied load rises. As the amount of silica gel in wt% increases (SG from 80% to 95%), the width increases, which indicates less resistance to the respective applied load. More specifically, the NSG80 and NSG85 batches show almost a similar value of scratch resistance to the respective applied load. These two composite batches can withstand a scratch load of up to 1300 g and with this, the load damaged width could be around 1100 µm. On the other hand, as evidenced by Figure 2, the steeper slopes of the higher filler batches (NSG90 and NSG95) indicate the increased sensitivity of the composite coating to rising scratch loads. This is demonstrated by the scratch widths of 1900 µm and 2400 µm for a 700 g (NSG90) and 500 g (NSG95) scratch load, respectively. This lower strength at higher levels (e.g., NSG95) is caused by the lower amount (5 wt%) of polymer that acts as a binder for SG particles. Anyway, this scratch resistance is consistent with results reported in the literature for similar coatings [17].

Furthermore, this test offers crucial insights into the composite coating’s shear strength and its overall mechanical stability. Two properties are paramount: the interfacial shear strength (cohesive bonding) between the filler and polymer matrix, and the adhesion (adhesive bonding) between the coating and substrate. Stronger interfacial bonding enhances stress distribution across the silica gel–polymer interface, which, in turn, improves both shear strength and scratch resistance. The composites with lower filler content (80 and 85 wt%) demonstrate higher shear strength, likely because a greater polymer content strengthens interparticle bonds. At the same time, concerning composite coatings with large filler content (90 and 95 wt%), the cohesive properties of the matrix within the SG particles are significantly reduced and this leads to easy crack formation and propagation upon the application of external force by the indenter.

**Figure 3:**
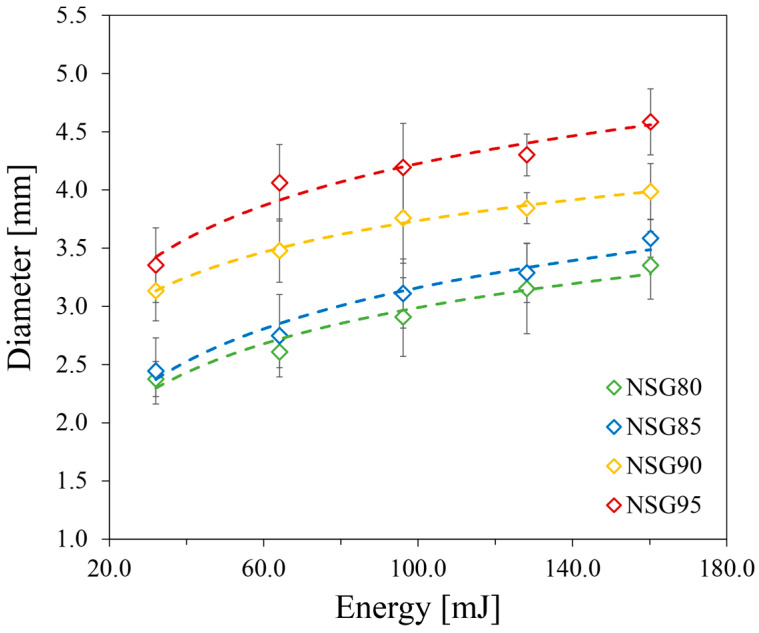
Impact diameter evolution at increasing impact energy for all composite coatings during impact test. The experimental data were fitted using logarithmic curves (dashed lines) yielding R^2^ ≥ 0.95 for all tested samples. Error bars represent the standard deviation of three independent measurements.

The impact test provides another valuable perspective, offering an indirect assessment of the composite coatings’ durability against sudden mechanical shocks. This test measures the coatings’ resistance to cracking under sudden mechanical stress, providing insight into its notch toughness and energy absorption capacity. Figure 3 shows the impact diameter’s evolution with increasing impact energy for all silica gel contents. The test directly correlates the absorbed energy of each sample batch to its resulting damaged area. The test was not performed on the unfilled Nexar sample due to its reduced coating thickness, which would compromise the validity of the measurement.

All batches exhibited consistent impact resistance, with the damage diameter increasing logarithmically as impact energy rose. As shown in Figure 3, the NSG80 and NSG85 composite coatings showed a quite smaller damage area (2.4 mm diameter) compared to the other batches. These performances can be attributed to their higher polymer content, which enhances the composite’s overall toughness and ductility.

Initially, the size of the damaged areas increases across all samples as impact energy rises.

However, a significant shift in behavior is evident: for the NSG80, NSG85, and NSG90 composites, the growth of the damage zone becomes less pronounced after an impact load of 96.1 mJ, with the maximum affected diameters remaining consistently between 3.0 and 3.5 mm. This suggests an energy dissipation mechanism that occurs beyond this critical load. Conversely, the NSG95 sample displays a steeper and more continuous rate of damage progression, leading to a significantly larger affected diameter of approximately 5.0 mm. This indicates a higher sensitivity to increasing impact energy, likely due to its lower polymer content impacting its ability to absorb and distribute stress effectively.

Despite these differences in damage size, a crucial and particularly positive finding emerges from these impact tests: even with localized damage present on the surface, the remainder of the coated composite remains firmly adhered to the aluminum substrate, showing no signs of delamination. This remarkable resistance to complete coating failure, coupled with the precise confinement of the damaged area, strongly suggests a suitable interaction among the Nexar matrix, the silica gel particles, and the underlying aluminum substrate. This cohesive and adhesive integrity is a key indicator of the composite’s overall reliability in demanding applications.

**Figure 4:**
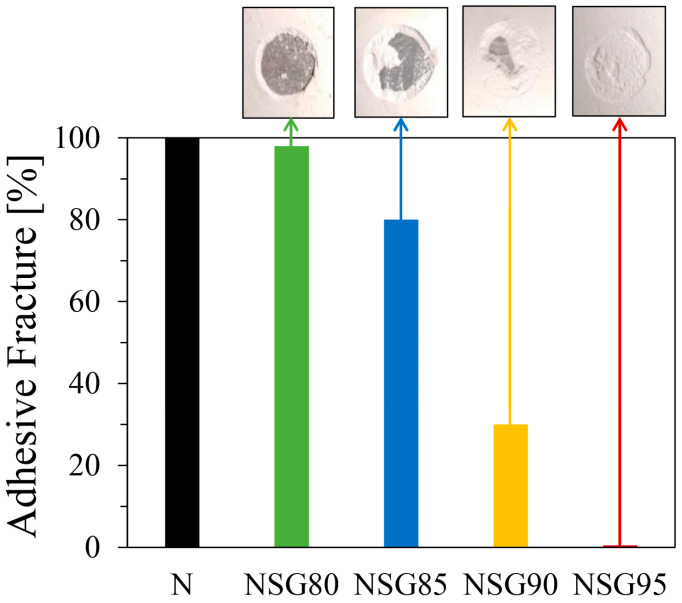
Adhesive fracture mechanism at varying filler contents for the composite coatings.

The pull-off adhesion strength of the composite coating on an aluminum substrate was assessed using a pull-off test, consistent with the procedure detailed in [36]. To comprehensively evaluate both the cohesive and adhesive properties of the coating, the resultant failure mode for all tested batches is evaluated in Figure 4.

With an increase in filler content from 80 to 95 wt%, the failure mechanism demonstrates a transition from a nearly adhesive to a cohesive mode. Specifically, in the NSG80 batch, the crack formation and propagation occur at the interface with the aluminum substrate, leading to a nearly 100% adhesive failure. This phenomenon strongly suggests that, at lower silica gel concentrations, the polymer matrix exhibits exceptional interaction with the silica gel particles, effectively transferring the applied stress directly to the substrate surface. In essence, the cohesive strength of the polymer–filler composite at this concentration is superior to the adhesive bond at the coating–substrate interface, leading to failure at the latter. Furthermore, observations regarding the adhesive fracture percentages for the NSG85 and NSG90 batches revealed values of approximately 80% and 30%, respectively. This indicates a significant adhesive failure mode for NSG85, suggesting a weaker bond at the coating–substrate interface, while NSG90 exhibits more balanced failure behavior. Conversely, the NSG95 batch presented a 100% cohesive failure mode. In this instance, the polymer matrix’s reduced ability to effectively transfer stress across the entire cross-sectional area of the composite coating becomes evident. This cohesive failure, occurring within the bulk material rather than at an interface, suggests that the bulk strength of the composite, particularly due to the diminished polymer binder content in NSG95, is the limiting factor. However, as the silica gel content increases to 95 wt% (NSG95), the limited polymer content compromises the cohesive strength of the matrix, leading to internal failure rather than interfacial detachment.

**Figure 5:**
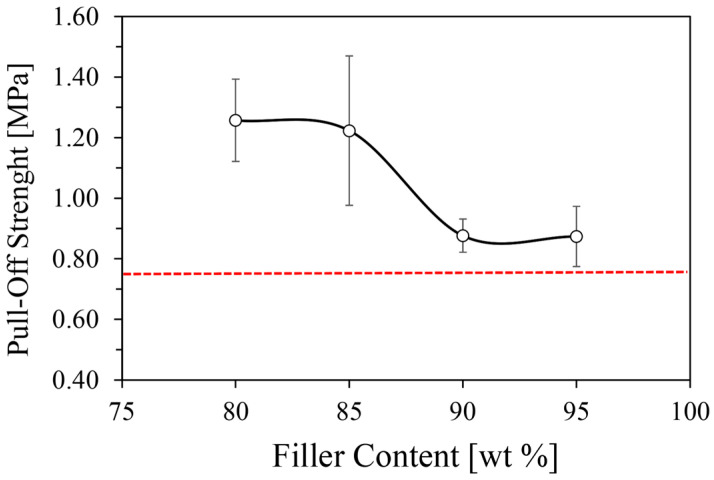
Pull-off adhesion strength of varying filler contents for the composite coating and minimum threshold (red line) for sorbent coatings [30]. Error bars represent the standard deviation of three independent measurements.

Figure 5 presents the adhesion strength trends for all tested composite coating batches, offering further insights into their adhesive properties.

The NSG80 and NSG85 batches showed good adhesion, exhibiting average pull-off adhesion strengths of 1.26 MPa and 1.36 MPa, respectively. These values signify a suitable bond with the substrate (the samples exhibited adhesive fracture), indicating that these compositions maintain strong interfacial integrity under tensile stress. Conversely, the NSG90 and NSG95 batches display a noticeable reduction in adhesion strength, averaging approximately 0.85 and 0.90 MPa, respectively. This represents a decrease of nearly 30% compared to the NSG80 batch, clearly highlighting the detrimental impact of excessive filler content on the overall adhesive and cohesive performance of the coating. This reduction is attributed to the diminished proportion of the polymer matrix, which is critical for establishing strong bonds both within the composite and at the coating–substrate interface, favoring a premature fracture by cohesive mode.

Interestingly, while the adhesion strength varies with filler content, the trends within batches of similar filler content (i.e., lower filler content batches NSG80/85 and higher filler content batches NSG90/95) do not show a significant impact on the interaction between the polymer matrix and the filler. However, a steeper slope transition from higher to lower adhesion strength is observed in coatings with higher filler content, further emphasizing the weakened adhesive and cohesive interaction due to the reduced amount of polymer matrix. Despite these differences, it is important to note that the adhesive strength achieved by all tested batches surpasses the minimum threshold of 0.75 MPa recommended for sorbent coatings, as indicated in [39]. This confirms that, even with increased filler content, the composite coatings retain sufficient mechanical integrity for their intended application.

Table 2 is presented to assess the performance of the developed silica gel composite coatings by offering a direct comparison with relevant findings reported in the existing literature. For this purpose, a standardized filler content of 90 wt.% was selected, enabling a consistent and meaningful evaluation against established benchmarks in the field.

When evaluating its scratch resistance, the silica gel/Nexar composite shows greater vulnerability to scratch loading compared to SAPO-34 and zeolite 13X-filled Nexar composites, exhibiting a 21.8% and 38.8% reduction, respectively. The decrease in scratch resistance becomes even more significant, approximately 75%, when benchmarked against SPEEK and S-rPEEK matrices. Regarding pull-off adhesion strength, the silica gel/Nexar composite performs 5.4% below the SAPO-34-based variant but surpasses the zeolite 13X-filled composite by 6.8%. This nuanced performance highlights areas of both strength and potential for improvement.

While the mechanical performance of the silica gel/Nexar composite may be lower than that of the higher-performance binders presented in Table 2, its industrial implications are nevertheless relevant. A major barrier to advancing sorption-based thermal energy management lies in the commercialization of composite coatings, primarily due to high manufacturing costs and the inherent complexity of applying these materials to sorption systems. Within this framework, this sulfonated polymer emerges as a more practical and readily available alternative compared to the binders previously reported. Despite its mechanical strength not matching the absolute highest-performing systems, it remains within the acceptable limits outlined in [42], making it a viable and scalable option for large-scale industrial applications. This balance between performance and practicality is relevant for real-world deployment.

### 3.2. Thermo-Physical Results

**Figure 6:**
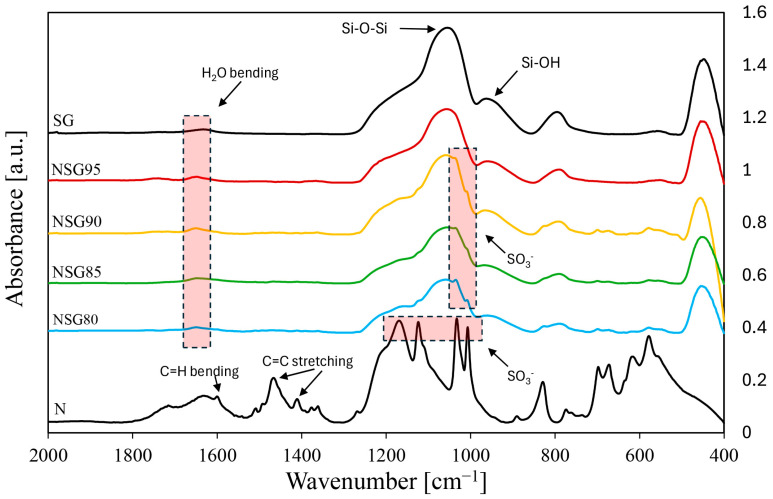
FTIR spectra of sulfonated polymer (N) pristine silica gel (SG) and related composites (NSG80-NSG95).

Figure 6 presents a comparative analysis of the FTIR spectra for composite coatings with varying contents of silica gel. For comprehensive reference, the spectra of both a blank sulfonated polymer and pristine silica gel powder are also included, allowing for the detection of subtle chemical changes and interactions within the composite structure.

For the Nexar polymer, several distinctive absorption bands were identified. A stretching vibration of the phenyl group appeared around 1631 cm^−1^, while the bending vibration of methylene (C-H) groups was detected at 1600 cm^−1^ [43]. The FTIR spectrum of raw Nexar provides further details: vibrational modes associated with C=C stretching within phenyl rings are evident at approximately 1400 and 1500 cm^−1^ and the distinctive presence of SO_3_^−^ functional groups is confirmed by absorption bands appearing at 1006, 1033, 1125, and 1165 cm^−1^ [44]. Moreover, the bands observed below 900 cm^−1^ are specifically assigned to the out-of-plane deformation of CH groups, characteristic of the polystyrene segment’s repeating units [45]. Concerning silica gel powder, a dominant peak at 1050 cm^−1^ corresponds to the asymmetric stretching of Si-O-Si bonds, while a peak at 940 cm^−1^ is attributed to Si-OH stretching, likely hydrogen-bonded to adsorbed H_2_O molecules [46]. Furthermore, the adsorption band at 1640 cm^−1^ is assigned to the bending vibration of water molecules [47]. Concerning the composite coating, due to the high filler content, the infrared spectra mainly display the characteristic peaks of silica gel. It is important to note, though, that in samples with lower filler content (ranging from 80 wt% to 90 wt%), polymer-related peaks are indeed visible. Specifically, signals at 1033–1006 cm^−1^, attributable to sulfonic bonds, can be observed, even with substantial overlap from the Si-O-Si signal of the silica gel. It is also possible to observe a low-intensity signal at 828 cm^−1^, which is assigned to the out-of-plane C-H deformation within the polystyrene component of Nexar.

**Figure 7:**
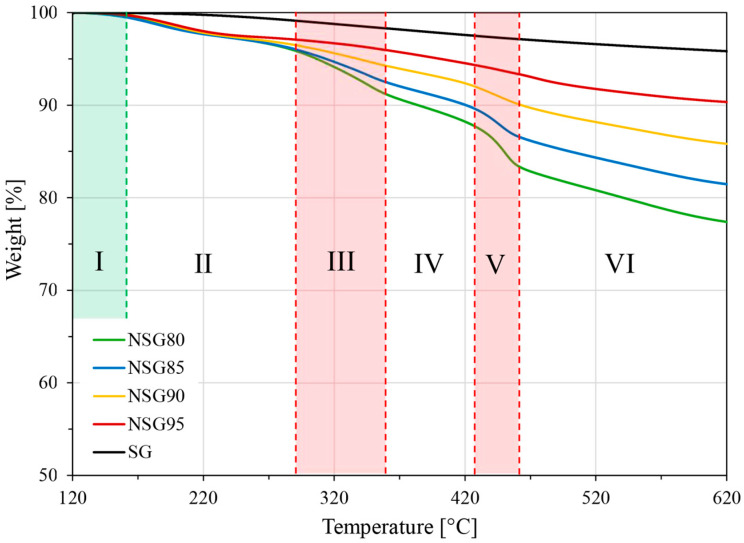
TGA plot of weight vs. temperature of all composite coatings and pure silica gel.

Thermogravimetric analysis (TGA), reported in Figure 7, was performed to investigate the thermal degradation behavior of the composite coating as the temperature increased up to 600 °C in air atmosphere.

The TGA curves for all composite coatings demonstrate a consistent thermal response, showing a similar pattern of weight loss regardless of silica gel content. Above 120 °C, thermogravimetric analysis (TGA) of the composite coatings reveals six distinct phases. The initial region I, extending up to approximately 160 °C, corresponds to the thermal stability region for both the polymer composite and pure silica gel, indicating that below this temperature, only volatile components (such as adsorbed water) are lost. Subsequently, from 160 °C to 280 °C (region II), a minimal weight loss is observed, which signifies the initiation of coating degradation within this temperature range, likely due to the loss of residual solvent or the breakdown of the least stable functional groups. Importantly, in this region, specifically above 220 °C, pure SG also begins its linear weight loss, a characteristic loss that continues almost linearly up to 620 °C and is typically attributed to the continuous dehydroxylation of the silica surface [48]. Moving to region III, spanning from 280 °C to 360 °C, the slope of the composite coating curve becomes significantly more pronounced. This accentuated degradation rate is directly related to the degradation of sulfonic acid groups (-SO_3_H), which are in the sulfonated polymer structure. The evidence supporting this assignment is the observation of a steeper slope for samples containing lower filler contents (e.g., NSG-80), as the polymer phase, where these groups reside, contributes a higher proportion to the overall mass loss. Another sharp decrease in weight is noted in region V, from 430 °C to 460 °C, ascribed to the onset of the main polymer backbone decomposition. Finally, regions IV and VI in the composite coating primarily reflect the linear weight loss behavior established in the pure SG, suggesting that the degradation profile in these zones is dominated by the filler component. As expected, the final weight loss is higher when the silica gel amount in the coating is lower, as the polymer phase is the primary source of mass loss. Crucially, the residual weight at 620 °C does not perfectly represent the content of silica gel in the coating. This discrepancy is due to the approximately 4% inherent weight loss observed for the pure silica gel itself over the full temperature range, primarily due to dehydroxylation.

**Figure 8:**
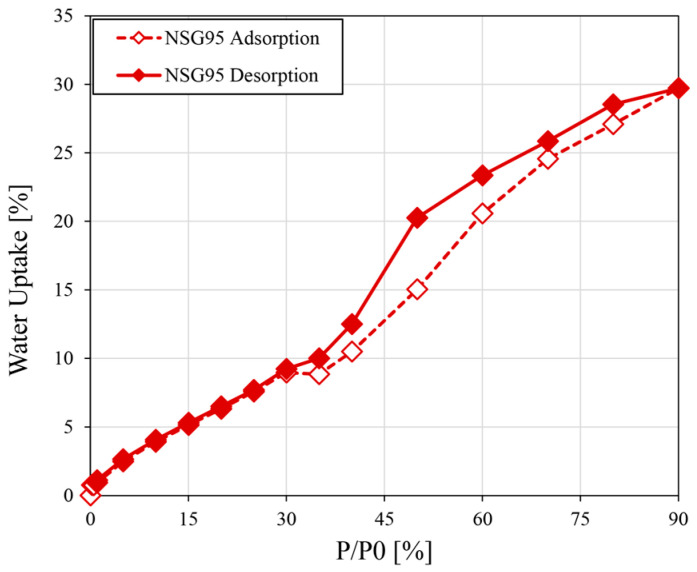
Water vapor adsorption and desorption isotherms at 30 °C for NSG95.

The performance of the adsorption/desorption process was assessed using isothermal analysis, as depicted in Figure 8. This figure illustrates the amount of adsorbate (water) retained per unit mass of the coating as a function of water vapor pressure, maintained at a constant temperature of 30 °C. The graph, reported for the NSG95 batch as reference, distinctly displays the behavior of both the adsorption phase (represented by empty markers and a dotted line) and the desorption phase (represented by solid markers and a solid line), allowing for a clear visual comparison of the uptake and release mechanisms.

The isothermal profile reveals discernible variations in water uptake and release by the composite material as relative humidity (RH) ranges from 30% to 90%. Notably, below 30% RH, both the adsorption and desorption curves exhibit a steady, nearly linear correlation between relative humidity and weight change. This characteristic behavior is indicative of monolayer adsorption at lower humidity levels, where water molecules primarily adhere to the external, readily accessible surfaces of the composite. In this region (P/P_0_ < 0.3), the amount of adsorbed water increases proportionally with the rising ambient moisture, suggesting a straightforward surface coverage mechanism. These interactions are predominantly weak physical adsorptions, leading to easily reversible uptake and, consequently, a non-hysteretic adsorption behavior in this initial region of the isotherm. As the relative vapor pressure (P/P_0_ > 0.3) progressively increases, the water molecules gain sufficient energy to penetrate deeper into the porous structure of the silica gel. This increased energetic state, coupled with the diminishing saturation deficit, facilitates the initiation of capillary condensation within the mesopores and micropores of the silica gel. This phenomenon leads to a marked and accelerated increase in the overall water uptake by the composite. Consequently, once this internal condensation occurs within the confined pore network, the subsequent desorption process follows a different pathway than adsorption. This divergence is attributed to the energy required to overcome capillary forces and empty these condensed liquid phases from the pores, often involving mechanisms like cavitation or inkbottle effects. This disparity between the adsorption and desorption paths is precisely what generates a noticeable hysteresis loop in the adsorption–desorption isotherm.

**Figure 9:**
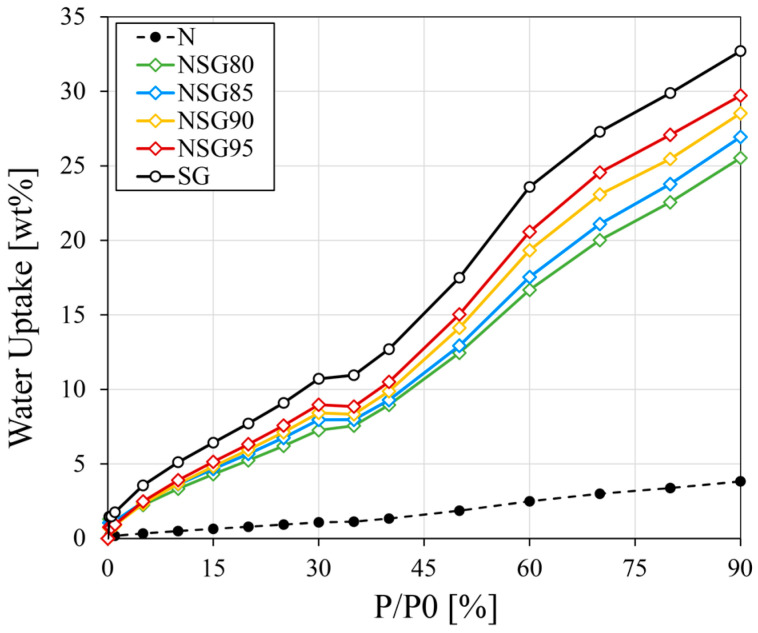
Adsorption isotherms in water vapor at 30 °C for all composite coatings. Pure silica gel powder (empty black circles) and neat sulfonated polymer (filled black circles) are included as references.

Figure 9 provides a comparative analysis of the isothermal adsorption curves for all composite coatings, specifically highlighting how adsorption capacity is affected by the variation in the silica gel content. The adsorption characteristics of pure silica gel powder (represented by empty black circle markers) and the neat polymer matrix (represented by filled black circle markers) are also included as references. Notably, the desorption isotherms for these composites exhibited trends consistent with those observed for NSG95 (previously detailed in Figure 8); therefore, they were omitted from this figure to maintain visual clarity and avoid overlapping data points.

The data clearly show that reducing the silica gel content directly lowers the overall adsorption capacity of the composite coatings. As expected, the NSG coatings consistently exhibit lower adsorption performances compared to the silica gel powder (SG sample). This is a direct result of the decreased proportion of active adsorbent material in each coating formulation. Among the various samples tested, the NSG95 batch demonstrated the most effective water uptake, reaching an adsorption value that was only approximately 9.1% lower than that of the pure silica gel powder (which achieved 32.7 wt%). Furthermore, the maximum adsorption capacities observed across all NSG batches, which range between 25.5 wt% and 29.7 wt%, are comparable to those of commercial adsorbent materials commonly used in water vapor sorption technologies [49].

**Figure 10:**
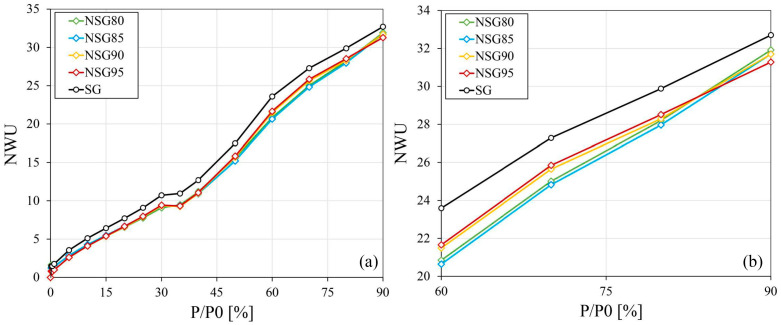
NWU values for all composite coatings: (**a**) full adsorption range; (**b**) magnification in the 60–90% P/P_0_ range.

Figure 10 also illustrates the trend of normalized water uptake (NWU), which is a key parameter used to assess the specific efficiency of the adsorbent phase within the composite. The NWU is determined by normalizing the total water uptake of the sample to the actual weight fraction of the filler (silica gel) present in the coating, according to the following equation:

(1) $$NWU=\;\frac{Water\;Uptake\;[wt\%]}{Filler\;content\;[wt\%]}$$

This calculation isolates the performance of the active filler from the inactive polymer matrix, allowing for a direct comparison of adsorption effectiveness across different filler loadings (80–95 wt.%).

The obtained curves are essentially superimposed, presenting a small reduction of approximately 4% in adsorption capacity across the entire range of partial pressures. This reduction appears to be independent of the polymer content in the coating (which varies between 5% and 20%). As demonstrated in Figure 10a, the NWU curves for all composite formulations are essentially superimposed, confirming that the polymer matrix content does not fundamentally alter the adsorption behavior of the active zeolite filler. To address the overlap of the curves and improve clarity, a magnification has been implemented within Figure 10b, focusing on the 60–90% P/P_0_ range, where the differences between the samples are most significant.

Additionally, the maximum normalized water uptake shows a reduction in adsorption capacity attributable to the presence of the polymer that ranges between 2.4% and 4.3% with respect to the pure sorbent. This means, for example, for the 90% formulation, which achieved a reduction of 3.1%, that 96.9 wt% of the silica gel in the composite coating actively participates in the adsorption and desorption processes.

The values of active sorbent obtained with this procedure are reported in Table 3 and compared with other adsorbent-based coatings in the literature. Among all the reported coatings, the SG/Nexar formulation achieved higher average values of active sorbent percentages across all the filler contents.

These results indicate a successful synergy between the components; the significant adsorption capacity proves that the silica gel remains fully functional despite being embedded in the polymer. This implies that the matrix acts as a facilitating medium rather than a diffusion barrier, allowing water vapor to interact freely with the adsorbent filler. Such behavior is essential for ensuring that the thermodynamic properties of the silica gel are fully utilized in the final coating for thermal management by sorption technology.

It effectively favors the water vapor flow, facilitating the adsorption and desorption phenomena in the silica gel sorbent. The polymer’s open and hydrophilic nature inherently supports this process, as it encourages water vapor permeation [28]. Consequently, the composite maintains high adsorption efficiency, ensuring that the silica gel retains its functionality in capturing and releasing water vapor across varying humidity and temperature conditions.

**Figure 11:**
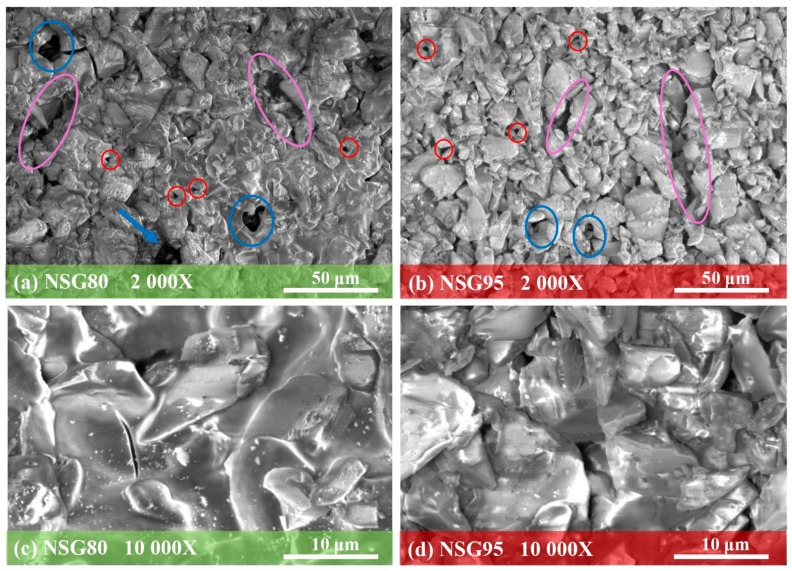
SEM micrographs at 2000× magnification for (**a**) NSG80 and (**b**) NSG95 and at 10,000× magnification for (**c**) NSG80 and (**d**) NSG95.

The surface morphology of the samples was examined using SEM at a magnification of 2000× and 10,000× to investigate the interfacial interactions between the Nexar polymer matrix and silica gel particles. Figure 11 presents the surface morphology of the composites containing 80 wt% and 95 wt% silica gel. In both cases, the filler particles appear densely packed and well-embedded within the Nexar polymer matrix, forming a uniform and cohesive structure.

At 2000× magnification, the coating displays intrinsic macro-channels (highlighted in pink in Figure 11a,b for both NSG80 and NSG95). These channels form naturally during the preparation process and act as preferential pathways for water vapor diffusion. Additionally, the presence of micro-voids (marked in red) and macro-voids (marked in blue) further enhances vapor transport throughout the composite coating, supporting efficient mass transfer during adsorption and desorption cycles. Thus, the intrinsic porous microstructure created by the high filler loading (80–95 wt.%) ensures that the silica gel active sites remain accessible. By correlating these chemical and structural attributes with the resulting adsorption kinetics and capacity, the overall material performance is comprehensively rationalized.

At 10,000× magnification (Figure 11c,d), the micrographs reveal clearer interfacial bonding between the silica gel particles and the Nexar polymer matrix, demonstrating strong integration and uniform dispersion.

The polymer’s influence is more pronounced in the NSG80 sample, where the higher polymer content results in well-encapsulated sorbent grains and a more interconnected polymer network. In contrast, NSG95 contains only 5 wt% polymer, making the polymer phase less visible; however, the silica gel particles remain well interconnected and securely embedded. Overall, these observations confirm Nexar’s strong film-forming ability and effective binding performance, enabling the composite to maintain structural cohesion and particle integration even under dynamic mechanical stresses.

These insights provide valuable confirmation regarding the thermal and sorption characteristics of the silica gel-based composite coating, emphasizing its promising suitability for the implementation of adsorption-based technologies (air conditioning, dehumidification, …). By examining the isothermal curves, a suitable indication is given of the material’s effective performance in terms of interaction with water vapor, thereby highlighting its capability to absorb and release water vapor efficiently. This functionality plays a key role in enhancing the overall thermal regulation mechanisms and driving the development of eco-friendly thermal management systems in the electric vehicles field.

## 4. Conclusions

This study comprehensively evaluated the mechanical integrity and sorption performance of novel silica gel/sulfonated polymer composite coatings on metallic substrates, quantifying their suitability for advanced thermal management in electric vehicles.

- Scratch resistance varied significantly with silica gel content: unfilled Nexar showed superior performance with minimal groove width, while composite coatings exhibited increasing width with load. Specifically, NSG80/NSG85 withstood 1300 g loads with ~1100 µm damage, whereas NSG90 showed 1900 µm damage at 700 g and NSG95 displayed 2400 µm damage at 500 g, a decline attributed to reduced polymer binder (down to 5 wt% in NSG95).
- The impact test showed consistent impact resistance across all batches, with damage diameter increasing logarithmically with impact energy. NSG80 and NSG85 exhibited superior performance with a 2.4 mm damage diameter. While all composites exhibited a continuous increase in damage, the progression for NSG80, NSG85, and NSG90 slowed after a 96.1 mJ load, remaining between 3.0 and 3.5 mm. Conversely, NSG95 had a steeper progression, reaching a 5.0 mm diameter. Crucially, all batches, including NSG95, maintained firm adhesion to the aluminum substrate without delamination.
- Pull-off adhesion strength measurements revealed strong adhesion for NSG80 and NSG85, yielding values of 1.26 and 1.36 MPa, respectively; these samples primarily exhibited adhesive failure. Conversely, NSG90 and NSG95 showed a nearly 30% reduction in adhesion strength, with values decreasing to 0.85 and 0.90 MPa, respectively. This drop was accompanied by a shift to 100% cohesive failure for NSG95, confirming the mechanical limitations of excessively high filler loadings. Despite this trend, all investigated batches exceeded the 0.75 MPa minimum threshold, demonstrating sufficient mechanical integrity for the target application.
- In terms of adsorption performance, the data consistently showed that reducing silica gel content led to a decline in overall adsorption capacity. The maximum adsorption capacities for the NSG batches, ranging between 25.5 wt% and 29.7 wt%, are comparable to commercial adsorbent materials. These values represent a minor reduction (between 9.1% and 22.0%) relative to the maximum capacity of the pure silica gel, a decrease that is strictly proportional to the polymer/filler ratio, suggesting that the sulfonated polymer matrix does not significantly impede water vapor diffusion. The observed thermal stability, with the polymer matrix maintaining integrity up to 280 °C and sulfonic group degradation beginning beyond 280 °C, further supports their suitability for sorption applications.

Ultimately, this comprehensive characterization confirms that the developed silica gel/sulfonated polymer composites offer a compelling balance of mechanical robustness, reliable adhesion to metallic substrates, and high adsorption efficiency. This synergistic combination of properties, achieved through readily available and economical materials, definitively positions them as highly promising and cost-effective solutions for advanced thermal management systems in electric vehicles, addressing a critical need for sustainable and efficient energy solutions in the automotive sector.

## Figures and Tables

**Table 1:** Sample identification codes and corresponding formulation details: wet mixture components, dry coating filler-to-matrix ratios, and measured coating thickness.

Sample Code	Slurry			Composite Coating		
	Nexar (wt%)	Silica Gel (wt%)	DMF (wt%)	Nexar (wt%)	Silica Gel (wt%)	Coating Thickness (mm)
N	20.00	0.00	80.00	100	0	0.20 ± 0.03
NSG80	5.00	20.00	75.00	20	80	0.76 ± 0.08
NSG85	4.00	22.67	73.33	15	85	0.92 ± 0.09
NSG90	2.86	25.71	71.43	10	90	0.80 ± 0.08
NSG95	1.54	29.23	69.23	5	95	0.91 ± 0.11

**Table 2:** Comparison of the mechanical properties among different sorbent-based composite coatings.

Coating Formulation	Scratch Test Groove Width at 1000 g [µm]	Pull-Off Test Adhesion Strength ^2^ [MPa]	Impact Test Imprint Diameter ^3^ [mm]
SG/Nexar	1944.5 ^1^	0.88	4.0
SAPO-34/Nexar [40]	1520.8	0.93	4.1
Zeolite 13X/Nexar [41]	1200.0	0.82	3.3
SAPO34/S-PEEK [36]	457.9	1.66	-
SAPO34/S-rPEEK [36]	484.0	2.05	-
SAPO34/Graphite/S-PEEK [31]	529.0	1.56	-

^1^ Groove width obtained with a 700 g applied load. ^2^ Minimum adhesion strength threshold reported in the literature: 0.75 MPa [39]. ^3^ Impact diameter obtained with a 160.2 mJ impact energy.

**Table 3:** Comparison of the percentage of active sorbent in the composite coating among different sorbent-based formulations.

	Percentage of Active Sorbent in the Coating [%]			
Sorbent Amount [wt%]	80	85	90	95
^1^ SG/Nexar	97.6	96.9	96.9	95.7
^2^ SAPO-34/Nexar [40]	80.5	86.7	92.2	97.1
^2^ 13X/Nexar [41]	92.3	94.5	96.5	98.2
^2^ SAPO34/S-PEEK [29]	95.0	96.1	97.1	96.9
^2^ SAPO34/Graphite/S-PEEK [31]	-	-	93.5	-

^1^ Isotherm at 30 °C, ^2^ Isobar at 11 mbar.

## Data Availability

The original contributions presented in this study are included in the article. Further inquiries can be directed to the corresponding author.

## Abbreviations

The following abbreviations are used in this manuscript:

EVs	Electric Vehicles
HVAC	Heating, Ventilation, and Air Conditioning
DCHE	Desiccant-Coated Heat Exchanger
AC	Air Conditioning
DCHP	Desiccant-Coated Heat Pump
MOFs	Metal–Organic Frameworks
S-PEEK	Sulfonated Polyether-Ether Ketone
FTIR	Fourier-Transform Infrared
TGA	Thermogravimetric Analysis
N,N-DMF	N,N-Dimethylformamide
RH	Relative Humidity
NWU	Normalized Water Uptake
